# MgIG exerts therapeutic effects on crizotinib‐induced hepatotoxicity by limiting ROS‐mediated autophagy and pyroptosis

**DOI:** 10.1111/jcmm.17474

**Published:** 2022-07-19

**Authors:** Min Li, Chenxiang Wang, Zheng Yu, Qin Lan, Shaolin Xu, Zhongjiang Ye, Rongqi Li, Lili Ying, Xiuhua Zhang, Ziye Zhou

**Affiliations:** ^1^ Department of Pharmacy The First Affiliated Hospital of Wenzhou Medical University Wenzhou China; ^2^ Department of Pathology The First Affiliated Hospital of Wenzhou Medical University Wenzhou China; ^3^ Clinical Research Center The First Affiliated Hospital of Wenzhou Medical University Wenzhou China

**Keywords:** crizotinib, hepatotoxicity, reactive oxygen species, autophagy, pyroptosis, magnesium isoglycyrrhizinate

## Abstract

Crizotinib (CRIZO) has been widely employed to treat non‐small‐cell lung cancer. However, hepatic inflammatory injury is the major toxicity of CRIZO, which limits its clinical application, and the underlying mechanism of CRIZO‐induced hepatotoxicity has not been fully explored. Herein, we used cell counting kit‐8 assay and flow cytometry to detect CRIZO‐induced cytotoxicity on human hepatocytes (HL‐7702). CRIZO significantly reduced the survival rate of hepatocytes in a dose‐dependent manner. Furthermore, the reactive oxygen species (ROS) assay kit showed that CRIZO treatment strongly increased the level of ROS. In addition, CRIZO treatment caused the appearance of balloon‐like bubbles and autophagosomes in HL‐7702 cells. Subsequently, Western blotting, quantitative real‐time PCR and ELISA assays revealed that ROS‐mediated pyroptosis and autophagy contributed to CRIZO‐induced hepatic injury. Based on the role of ROS in CRIZO‐induced hepatotoxicity, magnesium isoglycyrrhizinate (MgIG) was used as an intervention drug. MgIG activated the Nrf2/HO‐1 signalling pathway and reduced ROS level. Additionally, MgIG suppressed hepatic inflammation by inhibiting NF‐κB activity, thereby reducing CRIZO‐induced hepatotoxicity. In conclusion, CRIZO promoted autophagy activation and pyroptosis via the accumulation of ROS in HL‐7702 cells. MgIG exerts therapeutic effects on CRIZO‐induced hepatotoxicity by decreasing the level of ROS.

## 1 INTRODUCTION

Anaplastic lymphoma kinase (ALK) rearrangement is a fusion between the ALK gene and the echinoderm microtubule‐associated protein‐like 4 (EML4) gene. Molecular abnormality of the ALK gene is associated with permanent cell proliferation, leading to the growth of multiple tumours, such as non‐small‐cell lung cancer (NSCLC). Crizotinib (CRIZO) is an ALK inhibitor used in the treatment of advanced ALK‐positive NSCLC. It acts by competitively binding to the adenosine triphosphate (ATP) site of ALK and is effective in inhibiting aberrant ALK activation. Since approved by the US Food and Drug Administration (FDA) for NSCLC treatment in 2011,^1^ CRIZO has been used worldwide for several years with favourable clinical outcomes. However, in some large clinical trials, as high as 57% of patients receiving a standard dose of CRIZO had elevated aminotransferase (aspartate/alanine) levels, discontinuation of the drug was required in about 2%–4% of patients and mortality was reported in 0.1% of patients.^2^, ^3^, ^4^ Currently, effective approaches for preventing and treating CRIZO‐induced hepatotoxicity remain poorly understood as the underlying mechanism of CRIZO‐induced hepatotoxicity is yet to be fully elucidated.

Drug‐induced liver injury (DILI) is often complex and driven by multiple mechanisms, including apoptosis, autophagy, necrosis and oxidative stress. Prior studies evaluating the toxic effects of CRIZO on the liver have primarily focused on oxidative stress and mitochondrial apoptosis.^5^, ^6^, ^7^ Besides, although substantial necrosis was identified in CRIZO‐treated hepatocytes, the specific necrotic pathway mainly contributing to CRIZO‐induced hepatotoxicity remains controversial. Moreover, previous evidence has suggested that ALK inhibitors could induce autophagy in tumour cells by inhibiting mTOR phosphorylation, and this autophagy led to cancer cell death.^8^, ^9^, ^10^, ^11^ Thus, the present study also sought to determine whether autophagy is involved in CRIZO‐induced hepatotoxicity and explore other mechanisms associated with hepatotoxicity.

The primary principles for DILI treatment in clinical settings are stopping the drug on time and treating DILI with appropriate anti‐inflammatory and hepatoprotective agents based on the clinical patterns of DILI. Liver transplantation is recommended if DILI damage is severe and irreversible.^12^, ^13^ Because DILI pathogenesis is complex, no specific drugs are currently available. Magnesium isoglycyrrhizinate (MgIG) is a novel α‐isomer compound synthesized by isomerization and salification from 18β‐glycyrrhizic acid, a triterpenoid extracted from Glycyrrhiza glabra. It has been reported to exhibit antioxidant, anti‐inflammatory and anti‐allergic pharmacological activities and is used for the treatment of viral hepatitis and abnormal liver function.^14^, ^15^, ^16^ Herein, we explored the effects and the underlying mechanisms of MgIG on CRIZO‐induced hepatotoxicity.

## 2 MATERIALS AND METHODS

### 2.1 Drugs and reagents

CRIZO was purchased from Energy Chemical Reagent Co., Ltd. (Shanghai, China), it dissolved in dimethyl sulfoxide (DMSO) to make a 20 mM stock solution and further diluted to desired concentrations with culture medium. Bafilomycin A1 (Baf A1) and N‐acetyl‐L‐cysteine (NAC) were purchased from MedChemExpress Co., Ltd. (New Jersey, United States). Baf A1 was dissolved in DMSO to make a 1 mM stock solution, which was diluted with culture media to the 5 and 10 nM concentration during experiments. NAC was straightly dissolved in culture medium to make a 10 mM solution. MgIG (5 mg/ml) was purchased from Zhengda Tianqing Pharmaceutical Group Co., Ltd. (Jiangsu, China).

### 2.2 Cell culture

HL‐7702 cells were obtained from the College of Pharmacy, Wenzhou Medical University. Hepatocytes were cultured in Roswell Park Memorial Institute (RPMI) 1640 medium (Gibco, China) supplemented with 10% foetal bovine serum (FBS) (Gibco, China) and 1% penicillin/streptomycin (Gibco, China) in a humidified incubator with 5% CO_2_ at 37°C. HL‐7702 cells were treated with different concentrations of CRIZO for 24 h. Next, cells were pretreated with 1 mg/ml of MgIG for 1 h followed by 15 μM CRIZO for 24 h.

### 2.3 Cell viability assay

Cell viability was evaluated using cell counting kit 8 (CCK8) assay (MedChemExpress, New Jersey, United States). After 24 or 48 h of drug exposure, the supernatants were removed and then 100 μl of CCK8 (10 μl/100 μl culture medium) was added to each well. After 2 h incubation, the optical density (OD) was measured at a wavelength of 450 nm using a microplate reader (Thermo Fisher Scientific, Massachusetts, United States). Cell viability was calculated based on the absorbance values.

### 2.4 Cell death analysis

An Annexin V‐PE/7‐AAD apoptosis assay kit (Lianke Biotech, Hangzhou, China) was used to detect apoptosis and necrosis. After treatment with CRIZO for 48 h, hepatocytes were harvested and washed twice with pre‐chilled phosphate‐buffered saline (PBS). Then, the cells were resuspended in a 500 μl binding buffer containing 5 μl Annexin V‐PE and 10 μl 7‐AAD and incubated at room temperature in the dark for 5 min. After staining, cells were analysed using a flow cytometer.

### 2.5 Nuclear protein extraction

Nuclear/cytosol protein extraction was performed using a nuclear protein extraction kit (Beyotime, Shanghai, China), according to the manufacturer's instructions. Briefly, cells were lysed with cytoplasmic lysis buffer on ice and vortexed vigorously. The lysates were centrifuged for 5 min at 16,000 *g* to obtain the supernatant containing cytosolic proteins and the pellet containing the nuclei. Following the nuclei were resuspended in 50 μl of nuclear extraction buffer, the samples were vortexed vigorously for 30 s with maximum power and then cooled on ice for 1 min, which was repeated 15 times to ensure the nuclei were lysed adequately. Then the samples were centrifuged for 10 min at 16,000 *g* to obtain nuclear protein. The proteins were boiled with 5 × loading buffer and then stored at −20°C for further analysis.

### 2.6 Western blotting

Cells were lysed by whole‐cell lysis buffer (Beyotime, Shanghai, China) containing protease inhibitor (PMSF) and phosphatase inhibitor. Then, protein lysates (20–40 μg per sample) were separated using polyacrylamide gels. After electrophoresis, the resolved proteins were transferred to 0.2 μm polyvinylidene fluoride (PVDF) membranes (Millipore, Massachusetts, United States) and blocked in 5% skimmed milk for 2 h. The blot was incubated overnight with primary antibodies and subsequently with the secondary antibody for 1 h. Antibodies against the following proteins were used: GAPDH, β‐actin, NRF2, Histone‐H3 and LC3 (Proteintech, Wuhan, China); NF‐κB (P65), Phospho‐NF‐κB P65 (Ser536), mTOR and Phospho‐mTOR (Ser2448) (Cell Signalling Technology, Massachusetts, United States); and NLRP3, Caspase‐1, GSDMD, MLKL and MLKL (phosphor S358) (Abcam, Massachusetts, United States).

### 2.7 Immunofluorescence

Firstly, HL‐7702 cells were plated into 24‐well culture plates with cell‐climbing slices and treated as indicated. After fixing (4% paraformaldehyde), permeabilizing (0.5% Trition X‐100) and blocking (5% bovine serum albumin), cells were incubated with NF‐κB (P65) antibody overnight, and then with CoraLite594‐conjugated secondary antibody (Proteintech, Wuhan, China) in the dark for 1 h. Finally, cell nuclei were stained with DAPI and then observed with a fluorescence microscope (Leica Microsystems, Wetzlar, Germany).

### 2.8 Quantitative real‐time polymerase chain reaction (RT‐qPCR)

Total RNA was extracted from samples using an RNA simple Total RNA kit (Tiangen, Beijing, China). RNA was reverse transcribed to synthesize cDNAs using a RevertAid RT Reverse Transcription kit (Thermo Fisher Scientific, Massachusetts, United States). Subsequently, cDNA was quantified by qPCR using the SYBR‐Green qPCR master mix (Toroivd, Shanghai, China). PCR amplification was performed using the following thermocycling protocol: pre‐denaturation at 95°C for 1 min followed by 45 cycles of denaturation at 95°C for 10 s, annealing at 60°C for 30 s. Finally, CT values were output from the instrument. ΔCT = CT (target gene) − CT (internal reference), ΔΔCT = ΔCT (experimental group) − ΔCT (control group). The relative expression of target genes was calculated using 2^−ΔΔCT^. All primer sequences are shown in Table 1.

**TABLE 1:** Sequences of the primers used for quantitative real‐time PCR

Gene	Forward primer (5′‐3′)	Reverse primer (5′‐3′)
GAPDH	CCAGCAAGAGCACAAGAGGAAGAG	GGTCTACATGGCAACTGTGAGGAG
HO‐1	CTGCTCAACATCCAGCTCTTTG	ATCTTGCACTTTGTTGCTGGC
Nrf2	GCCAACTACTCCCAGGTTGC	GTGACTGAAACGTAGCCGAAG
IL‐6	TTCGGTCCAGTTGCCTTCTC	CTGAGATGCCGTCGAGGATG
IL‐1β	GGCTTATTACAGTGGCAATGAGGATGA	TGTAGTGGTGGTCGGAGATTCGTAG

### 2.9 Measurement of Caspase1 activity

Caspase‐1 activity was determined using a chromogenic substrate Ac‐YVAD‐pNA (Beyotime, Shanghai, China) according to the manufacturer's instruction. Briefly, treated cells were collected and lysed with a cell lysate to extract cellular proteins. After aspiration of an equal volume of protein supernatant, 2 mM Ac‐YVAD‐pNA was added and the mixture was incubated at 37°C for 2 h. Finally, caspase‐1 activities of the samples were measured using a microplate reader at 405 nm.

### 2.10 Transmission electron microscopy

Treated and untreated cells (control cells) were harvested from the culture dish using a cell scraper and fixed with 2.5% glutaraldehyde at 4°C overnight. After staining, the cell masses were dehydrated, embedded in epoxy resin and sectioned at a thickness of 1 μm. Finally, ultrathin sections were examined under a H‐7500 electron microscope (Hitachi, Tokyo, Japan).

### 2.11 Evaluation of mitochondrial respiratory chain complex I (RCC I) activity

RCC I activity was measured using the mitochondrial respiratory chain complex I activity assay kit (Solarbio, Beijing, China). Cells were collected and cleaved, and an appropriate amount of mitochondria isolation reagent was added (1 ml per 5 × 10^6^ cells); then, cell samples were homogenized with a glass homogenizer (30 strokes) under ice bath condition; next, cells homogenate were centrifuged at 600 *g* for 10 min at 4°C in order to remove cell debris and nuclei; the supernatant was then transferred into a new centrifuge tube before centrifuging at 11,000 *g* for 15 min at 4°C; then, the supernatant was discarded; the isolated mitochondria (the precipitation) were lysed using lysis buffer at 4°C for 20 min and centrifuged at 13,000 *g* for 15 min at 4°C to obtain mitochondrial proteins. Then, RCC I activity was assessed spectrophotometrically.

### 2.12 Intracellular reactive oxygen species (ROS) quantification assay

Intracellular ROS levels were determined by staining cells with 2′, 7′‐Dichlorodihydrofluorescein diacetate (DCFH‐DA, Beyotime, Shanghai, China). Firstly, the 10 mM DCFH‐DA was diluted in a medium without FBS to make a 10 μM DCFH‐DA working solution. Secondly, cells were washed with PBS and incubated with DCFH‐DA working solution at 37°C for 20 min. After washing thrice in a medium without FBS, cells were harvested and analysed using a flow cytometer.

### 2.13 Statistical analysis

Statistical analysis was performed using GraphPad Prism 7.0 (IBM, California, United States). The results were calculated using data from at least three independent experiments. Values are presented as mean ± SD. Comparisons between two groups were performed using a two‐tailed Student's *t*‐test, and *p* < 0.05 was considered statistically significant.

## 3 RESULTS

### 3.1 Effects of CRIZO on hepatocyte cells

**FIGURE 1:**
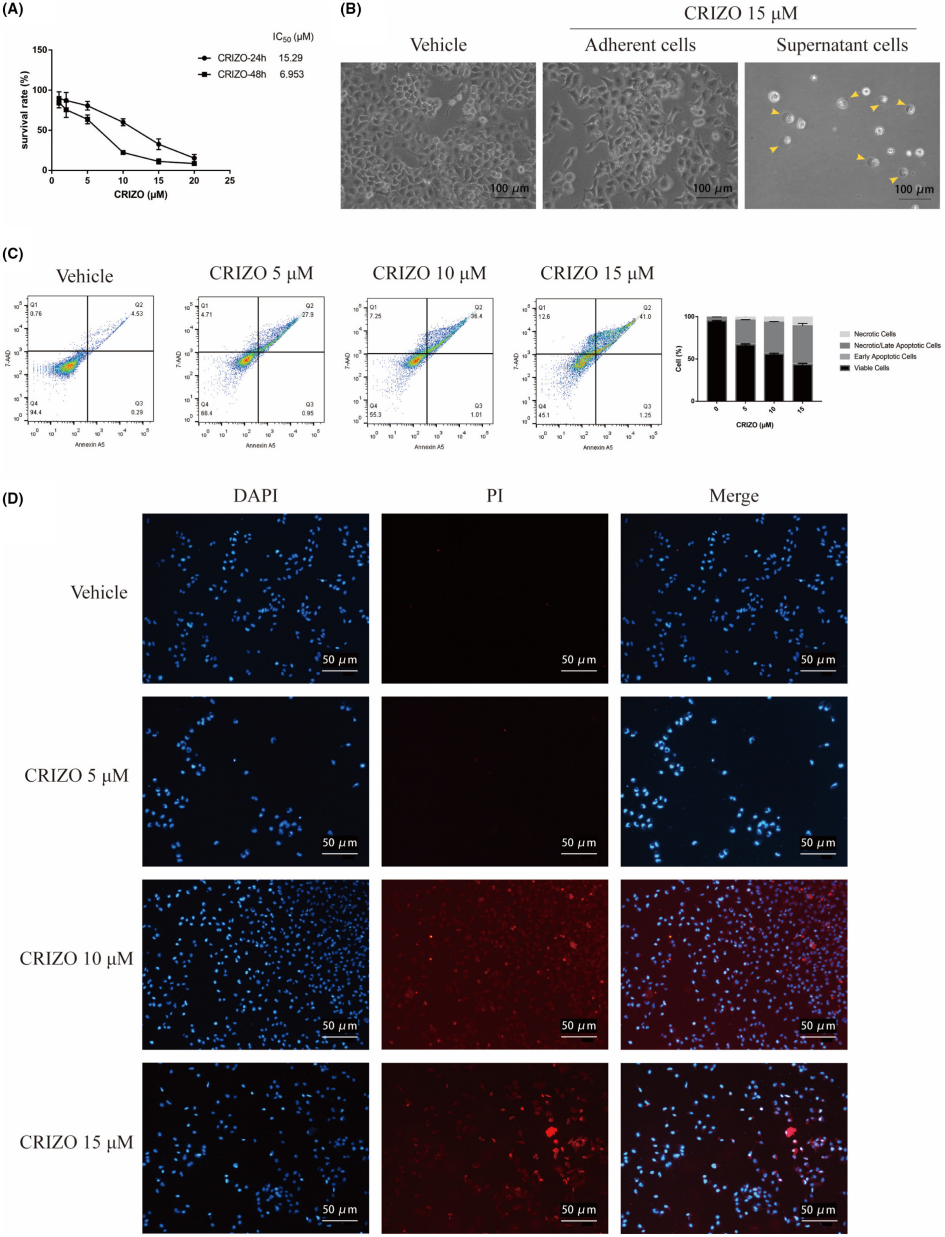
Characterization of hepatocyte death induced by CRIZO. (A) HL‐7702 cells were treated with different concentrations of CRIZO for 24 or 48 h. Cell viability was measured by CCK8 assay (*n* = 5). (B) Optical microscopy images of adherent cells and nonadherent cells treated with DMSO or CRIZO (scale bar: 100 μm). (C) HL‐7702 cells were treated with CRIZO (5–15 μM) for 48 h. The percentage of apoptotic and necrotic cells were determined by flow cytometric with Annxin A5‐7AAD staining (*n* = 3). Lower left quadrant, living cells; lower right quadrant, early apoptotic cells; upper left quadrant, necrotic cells; upper right quadrant, late apoptotic cells or necrotic cells. (D) Fluorescence images of DAPI/PI (scale bar: 50 μm). Necrotic cells were observed by fluorescence labelling with DAPI (blue) and PI (red).

As shown in Figure 1A, the survival rate of HL‐7702 cells reduced with an increase in CRIZO concentration and exposure time, with IC50 values of 17.2 μM for 24 h and 6.9 μM for 48 h, respectively, which suggested a great likelihood of hepatocellular damage during CRIZO administration. Hepatocytes were swollen and deformed with increased vacuolation of the cytoplasm 24 h after CRIZO treatment at 15 μM (Figure 1B). Interestingly, evident balloon‐like bubbles (arrows) were observed in the suspended dead cells and reminiscent of characteristic pyroptotic cell morphology (Figure 1B). Next, Annexin V‐PE/7‐AAD staining followed by flow cytometry was used to detect the proportion of apoptotic and necrotic cells. Treatment with CRIZO (5–15 μM) for 48 h increased the proportion of 7‐AAD‐positive cells (Figure 1C), which implied that CRIZO markedly induced liver cell necrosis. This was further confirmed by propidium iodide (PI) staining (Figure 1D). Overall, these findings indicated that CRIZO induced cell death by a necrotic mechanism.

### 3.2 CRIZO promoted hepatocyte pyroptosis and autophagy

**FIGURE 2:**
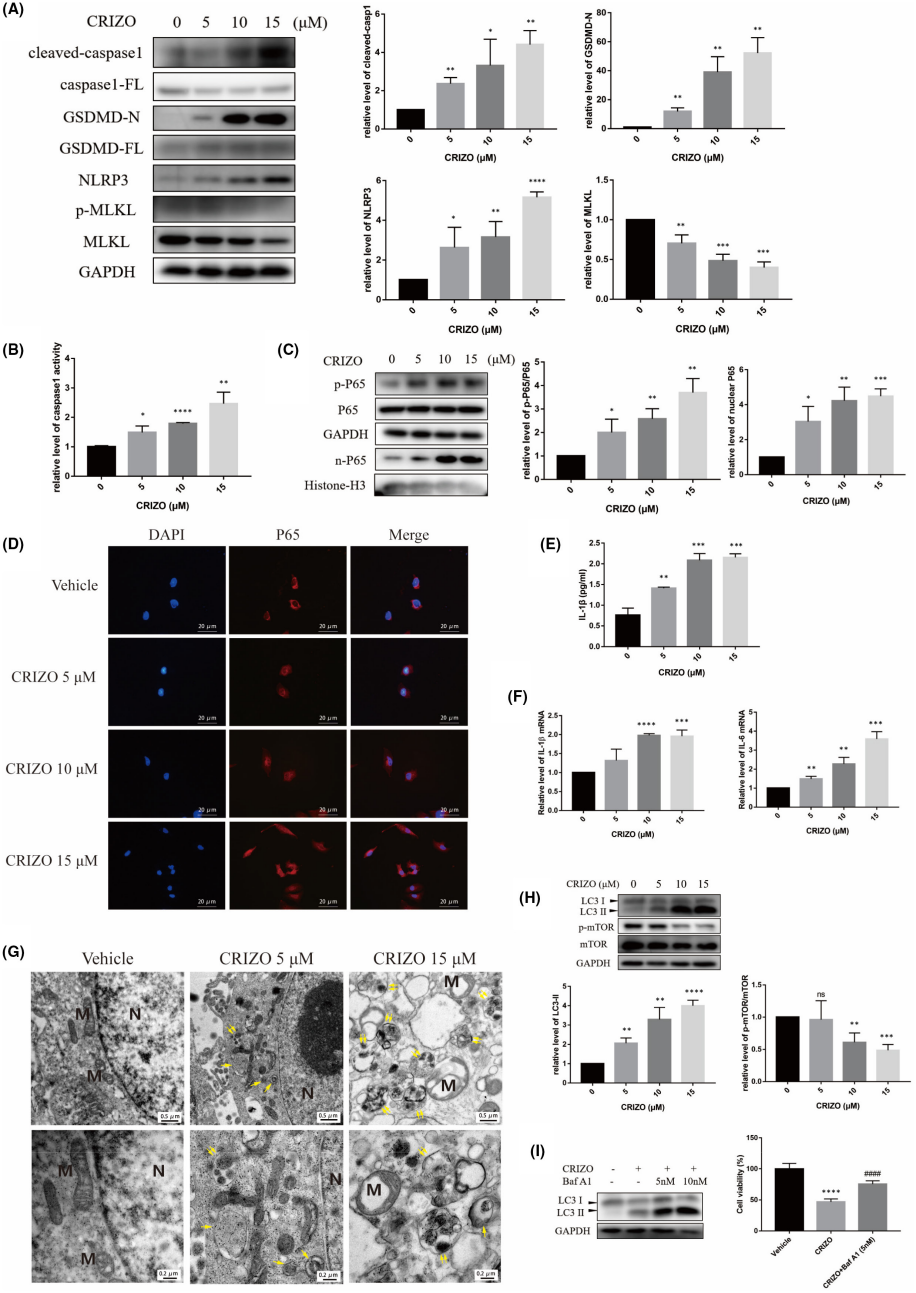
CRIZO promoted hepatocyte pyroptosis and autophagy. (A, G) After HL‐7702 cells were exposed to CRIZO (0–15 μM) for 24 h, the expression levels of cleaved‐caspase1, full‐length caspase1 (caspase1‐FL), GSDMD‐N, full‐length GSDMD (GSDMD‐FL), NLRP3, MLKL, phosphorylated MLKL, LC3, mTOR and phosphorylated mTOR in different groups were determined by Western blotting (*n* = 3). (B) HL‐7702 cells' caspase1 activity was determined after CRIZO administration for 24 h (*n* = 3). (C) The expression levels of phosphorylated P65 in whole cell and P65 in nucleus were determined by Western blotting (*n* = 3). (D) Nuclear translocation of P65 was observed by immunofluorescence labelling with DAPI (blue) and anti‐P65 (red). Scale bar: 20 μm. (E) IL‐1β secretion from HL‐7702 cells analysed by ELISA (*n* = 3). (F) The transcription levels of IL‐1β and IL‐6 were determined by qRT‐PCR. (G) The representative transmission electron micrographs of the HL‐7702 cells (*n* = 3). N, nucleus; M, mitochondrion; single arrow: autophagosome; double arrow: autophagolysosome; Scale bar in upper row images: 0.5 μm, scale bar in lower row images: 0.2 μm. (H) The expression levels of LC3 and phosphorylated P65 were determined by Western blotting. (I) HL‐7702 cells were treated by CRIZO with or without Baf A1 for 24 h, the levels of LC3 were determined by Western blotting (*n* = 3) and the cell viability was measured by CCK8 assay (*n* = 5). The data are expressed as the mean ± SD; *p < 0.05, **p < 0.01, ***p < 0.001 and ****p < 0.0001 vs. vehicle group; ^####^ *p* < 0.0001 vs. CRIZO 15 μM treatment group.

To further explore whether CRIZO promotes necroptosis, HL‐7702 cells were treated with CRIZO and the key regulator associated with necroptosis or pyroptosis was detected by Western blotting. As shown in Figure 2A, CRIZO treatment stimulated the upregulation of GSDMD‐N terminal (GSDMD‐N), NLRP3 and cleaved‐caspase‐1 but did not induce MLKL phosphorylation. CRIZO caused the downregulation of MLKL. CRIZO treatment also enhanced the activity of the caspase‐1 enzyme (Figure 2B). In addition, CRIZO effectively induced the secretion of IL‐1β and increased the mRNA level of IL‐1β and IL‐6 (Figure 2E,F). This suggested that CRIZO could activate pyroptosis through the classical pathway but not necroptosis in HL‐7702 cells. Moreover, whether CRIZO affected the activation of NF‐κB (P65) was investigated. As anticipated, CRIZO markedly promoted phosphorylation and nuclear translocation of P65 (Figure 2C,D), which favoured the formation of NLRP3 inflammasome.^17^, ^18^, ^19^, ^20^

Moreover, CRIZO treatment significantly increased autophagy in hepatocytes.

As shown in Figure 2G, CRIZO treatment induced mitochondrial swelling and formation of accumulated autophagosome and autophagolysosome structure in HL‐7702 cells; the autophagosome and autophagolysosome are important characteristics of autophagy. Consistently, Western blotting analysis demonstrated that CRIZO inhibited phosphorylation of mTOR and increased the level of LC3‐ΙΙ in HL‐7702 cells (Figure 2H). It was reported that sustained autophagy activation may promote necrosis in some cellular settings.^21^, ^22^, ^23^ Other studies reported that autophagy could be an adaptive response to protect cells under certain circumstances.^24^, ^25^, ^26^ Baf A1 is an autophagy inhibitor that can block autophagic flux by inhibiting both the formation of autophagolysosome and the degradation of LC3‐II. To determine the role of autophagy in CRIZO‐induced hepatocyte injury, the cell survival rate was evaluated in HL‐7702 cells that were exposed to CRIZO following Baf A1 pretreatment. CCK8 assay showed that Baf A1 successfully increased cell survival rate (Figure 2I). Thus, it is reasonable to deduce that autophagy contributed to CRIZO‐induced cytotoxicity in the liver.

Taken together, these results demonstrated that pyroptosis and autophagy collectively play a positive role in the development of hepatotoxicity induced by CRIZO.

### 3.3 CRIZO induced pyroptosis and excessive autophagy by accumulation of ROS

**FIGURE 3:**
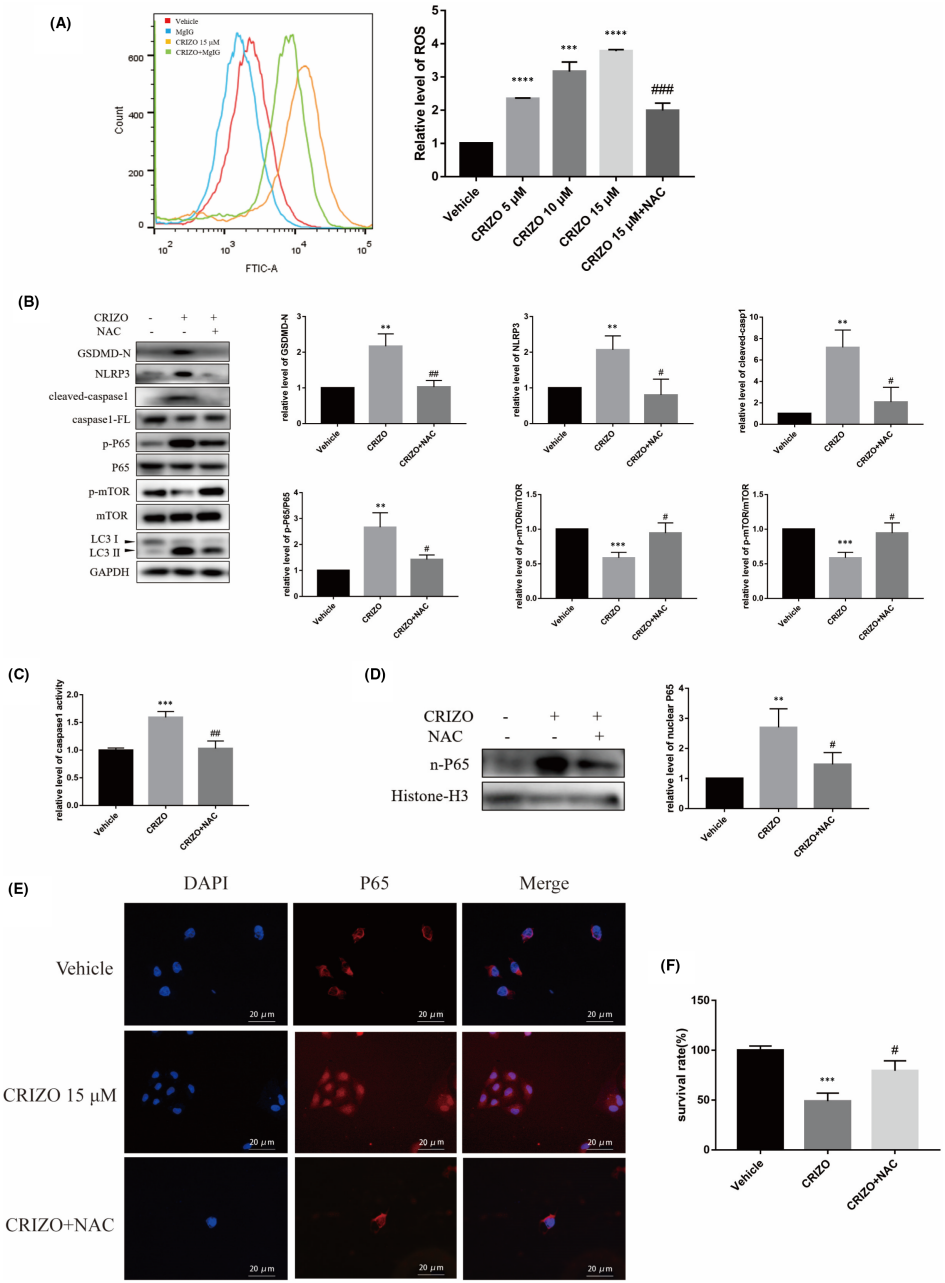
CRIZO induced pyroptosis and excessive autophagy by accumulation of ROS. (A) HL‐7702 cells were pretreated with or without 10 mM NAC before CRIZO treatment. The levels of ROS were determined by flow cytometric analysis in HL‐7702 cells (*n* = 3). (B) HL‐7702 cells were treated by 15 μM CRIZO with or without 10 mM NAC for 24 h. The expression levels of cleaved‐caspase1, caspase1‐FL, GSDMD‐N, NLRP3, LC3, mTOR, phosphorylated mTOR and phosphorylated P65 in different groups were determined by Western blotting (*n* = 3). (C) HL‐7702 cells' caspase1 activity was determined after administration for 24 h (*n* = 3). (D) The expression levels of P65 in nucleus were determined by Western blotting (*n* = 3). (E) Nuclear translocation of P65 was observed by immunofluorescence labelling with DAPI (blue) and anti‐P65 (red). Scale bar: 20 μm. (F) Cell viability was measured by CCK8 assay (*n* = 5). The data are expressed as the mean ± SD; *p < 0.05, **p < 0.01, ***p < 0.001 and ****p < 0.0001 vs. vehicle group; ^#^ *p* < 0.05, ^##^ *p* < 0.01, ^###^ *p* < 0.001 vs. CRIZO 15 μM treatment group.

ROS is a crucial molecule that regulates various cell death signalling pathways involved in DILI.^27^, ^28^, ^29^, ^30^, ^31^ Based on the phenomenon of mitochondrial swelling after CRIZO treatment (Figure 2G), we thought that CRIZO might result in mitochondrial damage and accumulation of ROS.^32^, ^33^ This conjecture was validated by detecting endocellular ROS at increasing concentrations of CRIZO (Figure 3A). Results were consistent with findings from the previous studies.^5^, ^7^

Next, the role of ROS in CRIZO‐induced hepatotoxicity was evaluated in vitro. Cells were pretreated with NAC (a potent ROS scavenger) for 1 h to counteract CRIZO‐induced ROS accumulation (Figure 3A), and then, cell viability and related protein levels were measured. On one hand, compared with the CRIZO group, the protein expressions of GSDMD‐N, NLRP3 and cleaved‐caspase1 were decreased in CRIZO plus NAC group (Figure 3B), and the activity of caspase1 was also decreased (Figure 3C). In addition, the interference of NAC caused reduction in phosphorylation and nuclear translocation of P65 (Figure 3B,D,E). On the contrary, the expression level of LC3 ΙΙ was decreased, and the level of p‐mTOR/mTOR was increased (Figure 3B). These results showed that reducing ROS accumulation in HL‐7702 cells alleviated CRIZO‐induced pyroptosis and excessive autophagy. Finally, as the CCK8 data shown in Figure 3F, NAC administration effectively recovered cell viability, suggesting that ROS accumulation significantly contributed to CRIZO‐induced hepatotoxicity.

### 3.4 MgIG reduced CRIZO‐induced ROS via upregulation of respiratory electron transport chain activity and re‐activation of Nrf2/HO‐1 pathway

**FIGURE 4:**
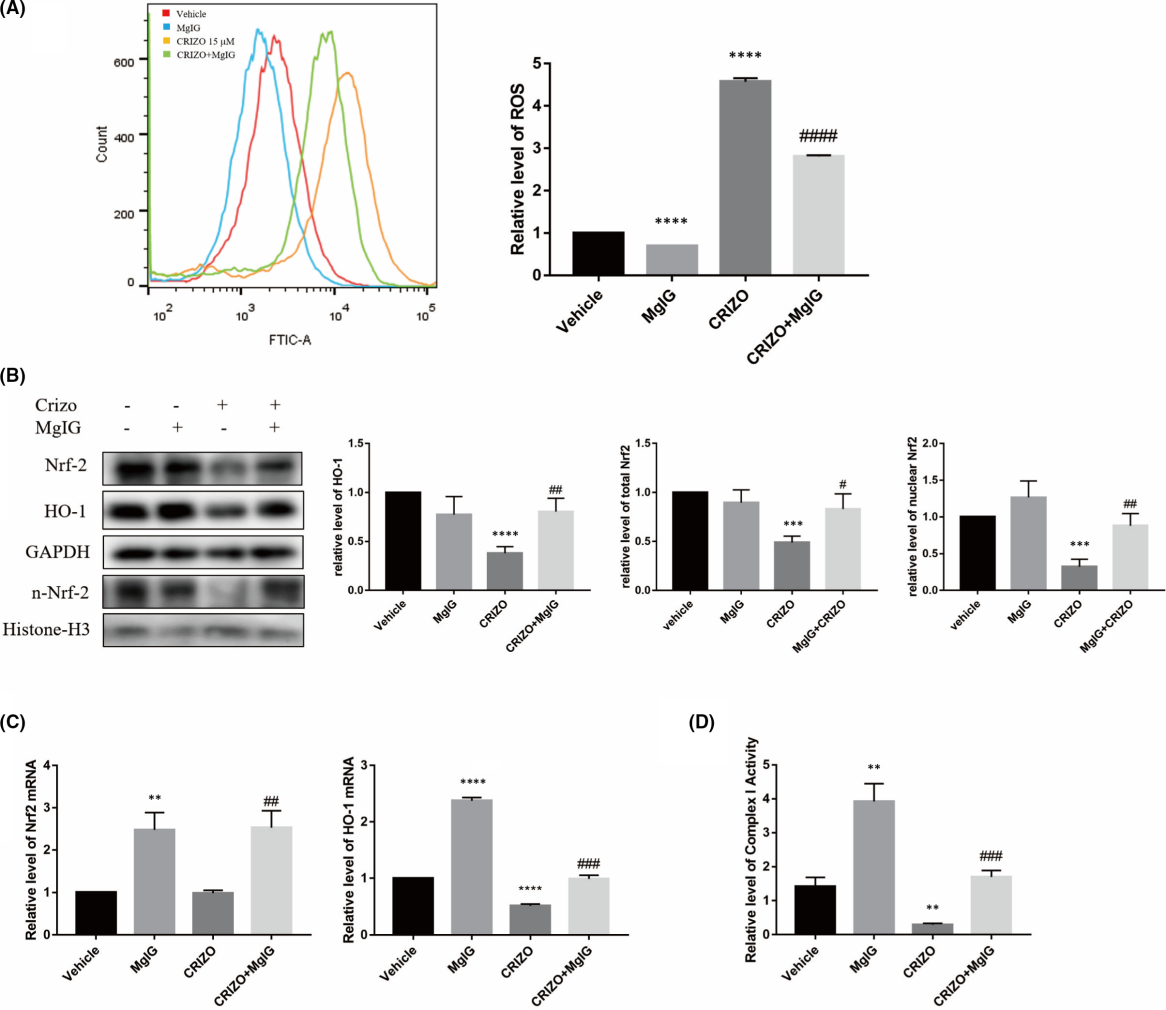
MgIG reduced CRIZO‐induced ROS via upregulation of respiratory electron transport chain activity and Nrf2/HO‐1 Pathway. (A) HL‐7702 cells were treated with 15 μM CRIZO or 1 mg/ml MgIG alone or in combination for 24 h. The levels of ROS were determined by flow cytometric analysis (*n* = 3). (B) The expression levels of HO‐1 and Nrf2 in whole cell and Nrf2 in nucleus were determined by Western blotting (*n* = 3). (C) The transcription levels of HO‐1 and Nrf2 were determined by qRT‐PCR (*n* = 3). (D) The activity of RCC Ι was determined by Mitochondrial Respiratory Chain Complexes Ι Activity Assay Kits (*n* = 3). The data are expressed as the mean ± SD; **p < 0.01, ***p < 0.001 and ****p < 0.0001 vs. vehicle group; ^#^ *p* < 0.05, ^##^ *p* < 0.01, ^###^ *p* < 0.001 and ^####^ *p* < 0.0001 vs. CRIZO 15 μM treatment group.

The modulatory role of ROS in CRIZO‐induced hepatotoxicity prompted us to seek an appropriate antioxidant for treatment. Acting as a hepatic protectant, MgIG presents significant advantages in the liver targeting distribution, time to effect, curative effect and safety.^34^ Furthermore, MgIG was found to maintain ameliorated oxidative stress and inflammation caused by several compounds, including epirubicin, arsenic trioxide, concanavalin A and lipopolysaccharide.^15^, ^16^, ^35^, ^36^, ^37^ Thus, it is most likely that MgIG can play an important role in reducing CRIZO‐induced hepatotoxicity. To support this hypothesis, we performed a series of experiments. HL‐7702 cells were pretreated with or without MgIG for 1 h and then treated with or without CRIZO for 24 h. Flow cytometry analysis of intracellular ROS showed that MgIG markedly reduced the CRIZO‐induced ROS accumulation (Figure 4A). CRIZO significantly decreased protein expression of HO‐1 and Nrf2 in whole cells as well as Nrf2 in the nucleus (Figure 4B), and co‐treatment of CRIZO with MgIG led to the re‐activation of the Nrf2/HO‐1 pathway. Moreover, RT‐qPCR results showed that the mRNA levels of Nrf2 and HO‐1 were significantly increased in the CRIZO plus MgIG group compared with CRIZO alone group (Figure 4C). Additionally, HO‐1 transcription was downregulated in the CRIZO group compared with the vehicle group (Figure 4C). Collectively, these results suggested that MgIG could reverse the inhibition of the Nrf2/HO‐1 pathway induced by CRIZO, which in turn decreased the accumulation of ROS. It is well known that impaired RCC Ι can cause severe electron leakage, which is a major source of mitochondrial ROS.^32^, ^38^ As anticipated, CRIZO decreased RCC Ι activity in hepatocytes, leading to excessive generation of ROS, and MgIG upregulated RCC Ι activity to reduce ROS (Figure 4D).

### 3.5 MgIG ameliorated CRIZO‐induced pyroptotic and autophagic hepatocyte damage

**FIGURE 5:**
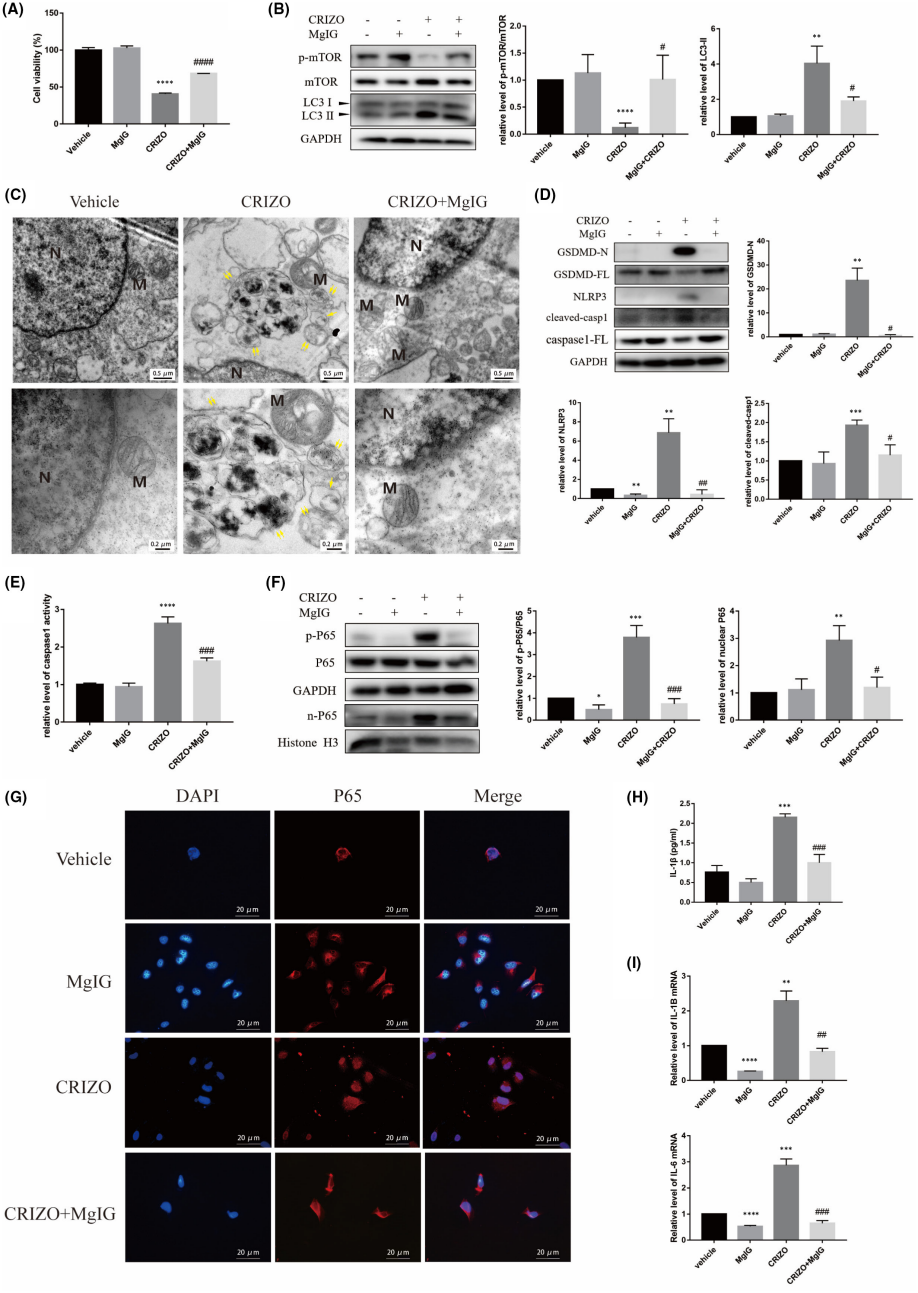
MgIG ameliorated CRIZO‐induced pyroptotic and autophagic hepatocyte damage. (A) Cell viability was measured by CCK8 assay (*n* = 5). (B) The expression levels of LC3, mTOR and phosphorylated mTOR in different groups were determined by Western blotting. (C) The representative transmission electron micrographs of the HL‐7702 cells. N, nucleus; M, mitochondrion; single arrow: autophagosome; double arrow: autophagolysosome. Scale bar in upper row images: 0.5 μm, scale bar in lower row images: 0.2 μm. (D) The expression levels of cleaved‐caspase1, caspase1‐FL, GSDMD‐N, GSDMD‐FL and NLRP3 in different groups. (E) Caspase1 activity was determined. (F) The expression levels of P65 in whole cell and P65 in nucleus were determined by Western blotting. (G) Nuclear translocation of P65 was observed by immunofluorescence labelling with DAPI (blue) and anti‐P65 (red). Scale bar: 20 μm. (H) IL‐1β secretion from HL‐7702 cells analysed by ELISA. (I) The transcription levels of IL‐1β and IL‐6 were determined by qRT‐PCR. The data are expressed as the mean ± SD; *p < 0.05, **p < 0.01, ***p < 0.001 and ****p < 0.0001 vs. vehicle group; ^#^ *p* < 0.05, ^##^ *p* < 0.01, ^###^ *p* < 0.001 and ^####^ *p* < 0.0001 vs. CRIZO 15 μM treatment group.

Supported by the above results, we further evaluated whether MgIG could protect hepatocytes from CRIZO‐induced pyroptosis and excessive autophagy. As anticipated, the CCK8 assay showed that the CRIZO plus MgIG group alleviated hepatic cell damage in comparison with the CRIZO alone group (Figure 5A). In addition, electron microscopy and Western blotting analysis revealed that MgIG partially normalized levels of autophagy altered by CRIZO (Figure 5B,C). MgIG markedly reversed the increase and activation of pyroptosis‐related proteins induced by CRIZO; these proteins included GSDMD‐N, NLRP3 and caspase1 (Figure 5D,E). The protein levels of p‐P65/P65 and nuclear P65 were reduced in cells treated by MgIG (Figure 5F,G). Subsequently, MgIG reduced the transcription and secretion of the inflammatory factors (Figure 5H,I).

## 4 DISCUSSION

CRIZO is one of the most potent drugs for ALK‐positive NSCLC, whose therapeutic efficacy is limited largely by hepatotoxicity. Previous developmental studies of CRIZO‐induced hepatotoxicity have mainly concentrated on mitochondrial apoptosis.^5^, ^7^ However, our study demonstrated that pyroptosis and autophagy are also involved in CRIZO‐induced hepatotoxicity.

Pyroptosis is inflammatory necrosis mediated by inflammasome activation. Compared with apoptosis, pyroptosis occurs more rapidly, which is accompanied with the release of inflammatory cytokines. The pyroptosis signalling pathway can be divided into classical and non‐classical pathways depending on whether caspase‐1 is involved or not. Cells promote inflammasome formation and activation after stimulation of the classical pathway, which cleaves and activates caspase‐1. The activated caspase‐1, in turn, cleaves and activates the downstream executioner Gasdermin (GSDM) family, including GSDMD and GSDME, to liberate the N‐terminal domain (GSDMD‐N/GSDME‐N). The N‐terminal domain strongly binds to inner leaflet lipids and forms pores of 10–15 nm of inner diameter, allowing the release of mature IL‐1β and triggering pyroptosis.^18^, ^39^, ^40^, ^41^ CRIZO and ceritinib have been reported to induce tumour cell pyroptosis via the caspase‐3/GSDME‐dependent signalling pathway.^40^ In our study, the expressions of NLRP3, cleaved‐caspase‐1 and GSDMD‐N were significantly upregulated in hepatocytes at 24 h after CRIZO administration. Additionally, transcription and secretion of IL‐1β were increased. Phosphorylation and nuclear translocation of P65 are implicated in NLRP3 inflammasome activation and inflammatory response regulation.^17^, ^18^, ^19^, ^20^ Our data showed that CRIZO treatment activated P65 in hepatic cells. These results demonstrated that CRIZO triggered hepatocyte pyroptosis via NF‐κB/NLRP3/GSDMD pathway. We also detected that CRIZO caused the downregulation of MLKL and did not induce MLKL phosphorylation. MLKL plays a critical role necroptosis via phosphorylated by RIP3. This suggested that CRIZO could not activate cell necroptosis.

Autophagy is a ‘self‐eating’ process negatively regulated by mTOR.^42^ DILI is often accompanied with the occurrence of autophagy.^43^ Our study found that CRIZO induced autophagic flux in hepatic cells in a dose‐dependent manner. However, the phosphorylation level of mTOR showed the opposite trend. Autophagy is known to be a ‘double‐edged sword’ as it is both a self‐defence mechanism against harmful stimuli and a programmed cell death mechanism.^42^, ^43^ For example, the activation of autophagy was conducive to protect against acetaminophen‐induced hepatotoxicity,^44^ but the gefitinib‐induced liver injury was considered to develop via autophagy^45^; in addition, some anticancer agents killed cancer cells by inducing autophagy.^8^, ^46^, ^47^ To determine whether autophagy plays a positive or a negative role in CRIZO‐induced hepatotoxicity, an autophagy inhibitor, Baf A1 was used to inhibit autophagosome‐lysosome fusion. The result showed that autophagy blockade ameliorated CRIZO‐induced hepatic cell injury, suggesting that excessive autophagy participated in hepatic cell death. However, the ‘crosstalk’ between autophagy and pyroptosis was not explored in the current study, which warrants further investigation.

Furthermore, the DCFH‐DA assay showed that the levels of intracellular ROS were markedly elevated after CRIZO treatment, which is consistent with previous reports.^5^, ^7^ A decrease in RCC Ι activity was also observed, which was the most important source of ROS. Mounting evidence has shown that ROS is one of the critical signal molecules of autophagy and pyroptosis.^17^, ^48^, ^49^, ^50^ Thus, NAC was used to partially scavenge ROS, and the results showed that ROS downregulation not only inhibited CRIZO‐triggered pyroptosis by preventing NF‐kB activation but also reduced autophagic flux via mTOR activation. This implies that CRIZO damaged the mitochondria leading to increased ROS level, which induced pyroptosis and autophagy to cause hepatotoxicity.

**FIGURE 6:**
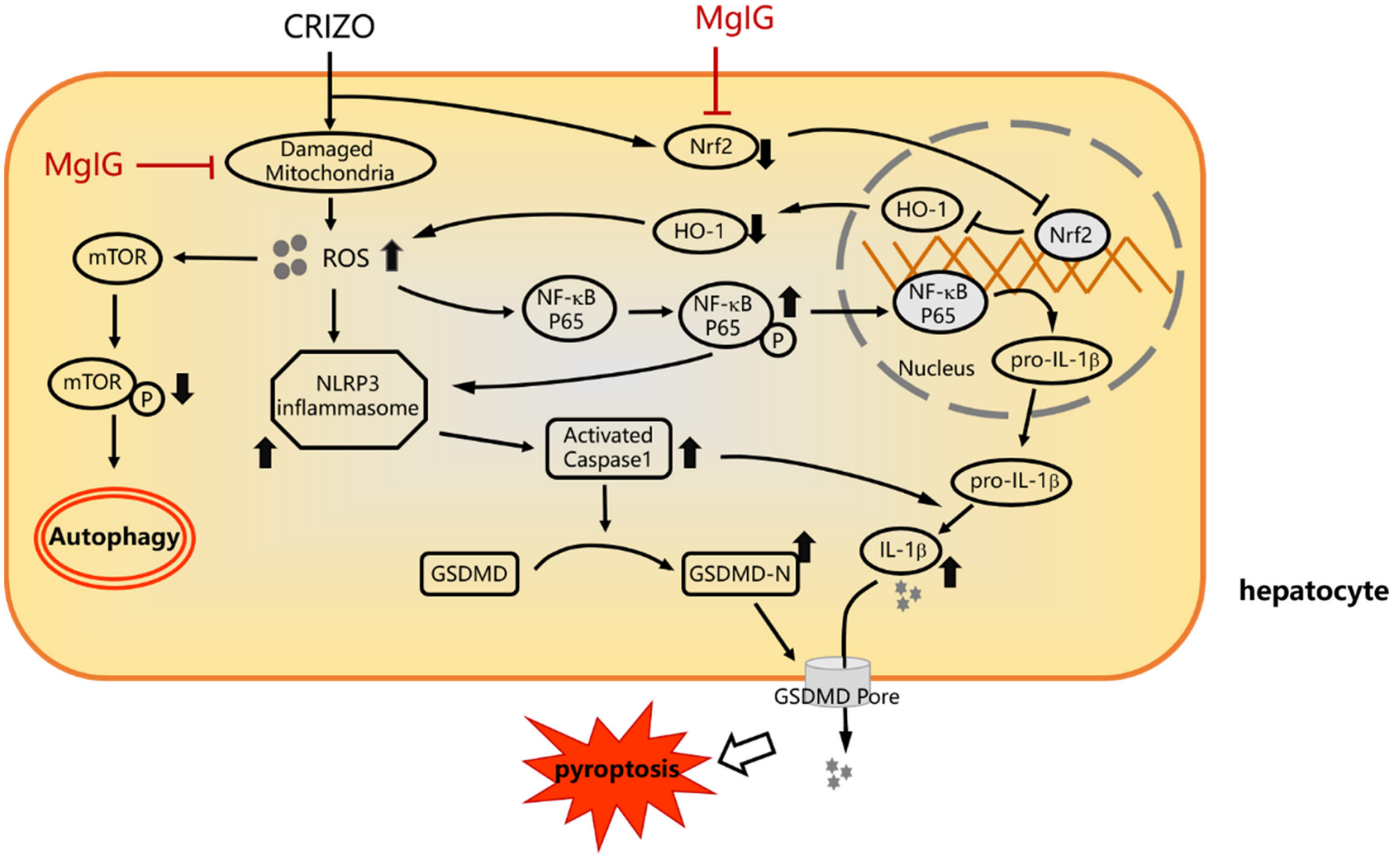
Intervention mechanism of MgIG on CRIZO‐induced hepatotoxicity. In hepatocytes, CRIZO activates excessive production of ROS due to RCC I dysfunction and Nrf2 downregulation, which causes the dysregulation of autophagy and activation of inflammasomes. The NLRP3 inflammasome activates caspase‐1, in turn cleaves GSDMD into GSDMD‐N, which facilitates the formation of membrane pores, ultimately resulting in pyroptosis. MgIG protected mitochondria from CRIZO damage and re‐activated Nrf2/HO‐1 antioxidant pathway, thus reduced CRIZO‐induced ROS accumulation, which in turn inhibits CRIZO‐induced autophagy and pyroptosis of hepatocyte.

The present study demonstrated that ROS is a positive regulator of CRIZO‐induced pyroptosis and autophagy in hepatocytes. The modulatory role of ROS in CRIZO‐induced hepatotoxicity prompted us to seek an appropriate antioxidant for treatment. MgIG is a strong hepatic protectant with anti‐inflammatory, antioxidant and hepatic targeting effects in vivo.^14^, ^15^, ^16^, ^34^ Although MgIG has broad application prospects in the clinic for DILI, the effectiveness and mechanism of MgIG in prophylaxis and treatment of CRIZO‐induced liver injury remain elusive. In this study, we verified that MgIG reduced CRIZO‐mediated ROS accumulation by re‐activation of the Nrf2/HO‐1 pathway and upregulation of RCC I activity, which in turn decreased CRIZO‐induced pyroptosis and autophagy, thereby enhancing the survival of hepatocytes. The intervention mechanism of MgIG on CRIZO‐induced hepatotoxicity is shown in Figure 6.

## 5 CONCLUSION

In summary, this study demonstrates a novel mechanistic basis for CRIZO‐induced hepatotoxicity, where ROS accumulation promotes pyroptosis and excessive autophagy. MgIG ameliorated CRIZO‐induced pyroptotic and autophagic hepatotoxicity by reducing ROS. These findings may form the basis for novel preventive and practical strategies for the clinical treatment of CRIZO‐induced hepatic injury.

## AUTHOR CONTRIBUTIONS

**Min Li:** Investigation (lead); writing – original draft (lead). **Chenxiang Wang:** Funding acquisition (equal); investigation (equal). **Zheng Yu:** Investigation (equal). **Qin Lan:** Investigation (equal). **Shaolin Xu:** Data curation (lead); formal analysis (equal). **Zhongjiang Ye:** Data curation (equal); formal analysis (lead). **Rongqi Li:** Data curation (equal). **Lili Ying:** Formal analysis (equal). **Xiuhua Zhang:** Project administration (equal); writing – review and editing (lead). **Ziye Zhou:** Funding acquisition (lead); project administration (lead).

## CONFLICT OF INTEREST

The authors declare that the research was conducted in the absence of any commercial or financial relationships that could be construed as a potential conflict of interest.

## Data Availability

The data that support the findings of this study are available from the corresponding author upon reasonable request.
